# Detection method of organic light-emitting diodes based on small sample deep learning

**DOI:** 10.1371/journal.pone.0297642

**Published:** 2024-02-05

**Authors:** Hua Qiu, Jin Huang, Yi-Cong Feng, Peng Rong

**Affiliations:** 1 School of Computing and Artificial Intelligence, Southwest Jiaotong University, Chengdu, China; 2 Information Center, Department of Natural Resources of Sichuan Province, Chengdu, China; 3 Archives Information Technology Division, Chengdu Bureau of Archives, Chengdu, China; Anhui University, CANADA

## Abstract

In order to solve the surface detection problems of low accuracy, low precision and inability to automate in the production process of late-model display panels, a little sample-based deep learning organic light-emitting diodes detection model SmartMuraDetection is proposed. First, aiming at the detection difficulty of low surface defect contrast, a gradient boundary enhancement algorithm module is designed to automatically identify and enhance defects and background gray difference. Then, the problem of insufficient little sample data sets is solved, Poisson fusion image enhancement module is designed for sample enhancement. Then, a TinyDetection model adapted to small-scale target detection is constructed to improve the detection accuracy of defects in small-scale targets. Finally, SEMUMaxMin quantization module is proposed as a post-processing module for the result images derived from network model reasoning, and accurate defect data is obtained by setting a threshold filter. The number of sample images in the experiment is 334. This study utilizes an organic light-emitting diodes detection model. The detection accuracy of surface defects can be improved by 85% compared with the traditional algorithm. The method in this paper is used for mass production evaluation in the actual display panel production site. The detection accuracy of surface defects reaches 96%, which can meet the mass production level of the detection equipment in this process section.

## Introduction

**Fig 1:**
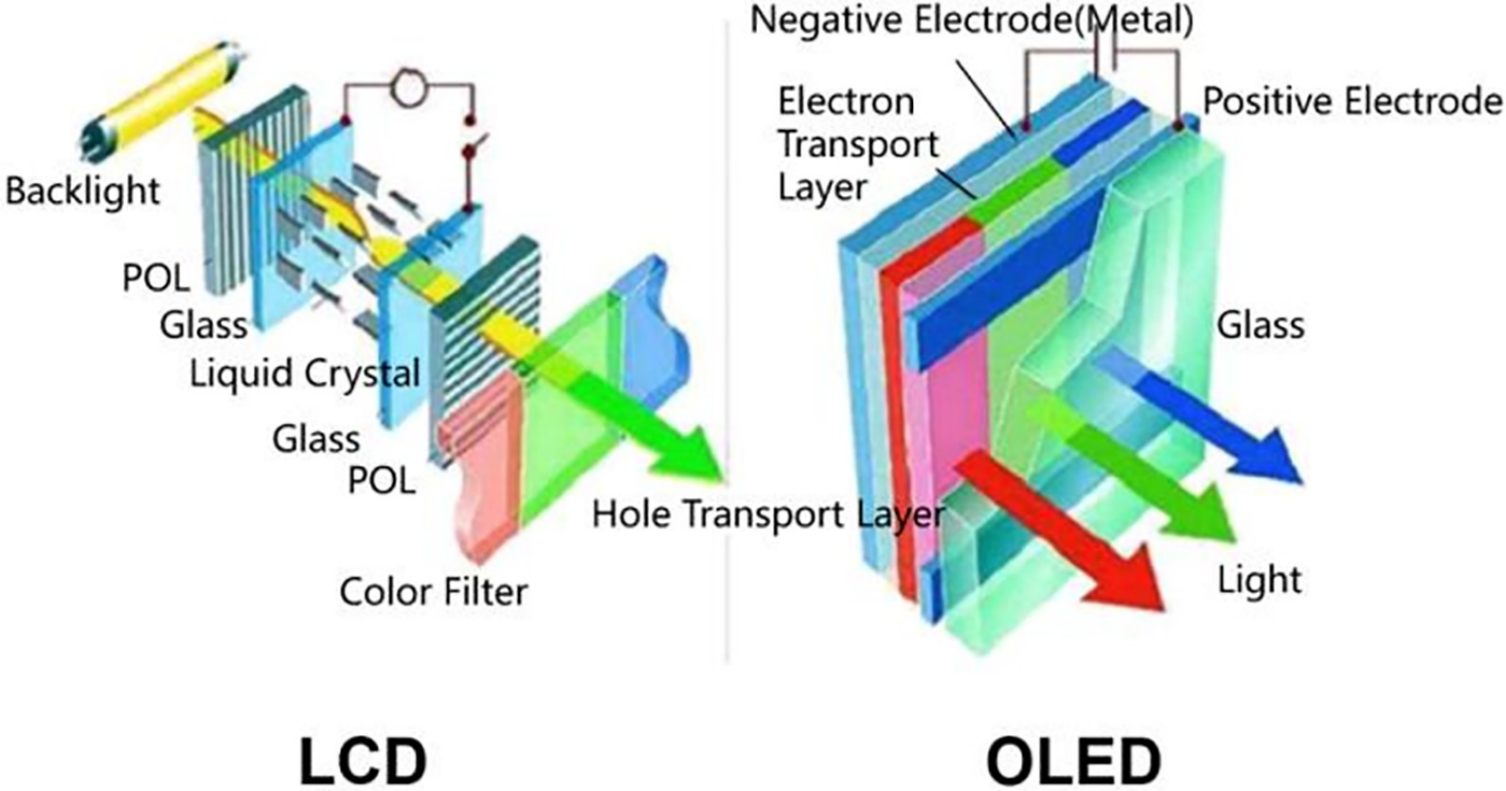
Luminous principle of LCD and OLED.

With the advent of the era of global 5g and Internet of everything, the rapid development of mobile streaming media and meta-universe has promoted the continuous upgrading and iteration of information display technology, and video data has become the main body of information flow [1]. In this wave of change in the display industry, OLED (organic light emitting diode) technology [2] is a new generation of mainstream flat panel display technology after the traditional liquid crystal display (LCD) technology. Compared with LCD display, OLED display has the advantages of light and thin appearance, energy saving, wide field of view, more realistic color saturation, high light and dark contrast, faster response time and high flexibility (its structure is shown in Fig 1).

Fig 1 displays the components of LCD and OLED. Both technologies have their strengths. OLED has superiority in contrast, viewing angles, black levels, and energy efficiency.

Due to the immature production technology of OLED, various defects such as point defects, line defects and mura defects occur in the process due to various factors such as foreign matters in the workshop environment, coating uniformity, film thickness differences, mask offset wear, CD changes, glass substrate defects and so on. These defects [3] occur frequently in the production process of OLED, affecting the delivery yield of products and causing a large amount of material waste. At present, the OLED manufacturing industry is facing the thorniest problem, which leads to the rise of processing costs.

In order to improve the automation level of the production line and the overall quality inspection level, general enterprises will introduce automated inspection machines in the form of assembly lines to replace manual inspection, so as to realize a low missed inspection, low over inspection, quantifiable and closed-loop machine vision inspection system [4]. Among the most important types of OLED image quality defects, the detection of point defects and line defects is relatively easy and quantifiable. Mura defect has become one of the defect types that are difficult to realize automatic detection in OLED panel image quality inspection due to its wide variety, low contrast, diverse shape and contour, and cannot be directly quantified.

The principle of machine vision automatic inspection is mainly to drive the product to light up a specific picture group, then use a high-resolution camera to collect the screen picture, send it to the algorithm for automatic analysis, and the results are fed back to the “programmable logic controller” PLC and the factory “manufacturing execution system” MES. The more difficult part is the development of the core detection algorithm, especially the mura automatic detection algorithm. In 1998, Pratt et al. first proposed the mura automatic detection algorithm system muralook [5], by combining various image processing operators, the system can screen and separate more than 20 different mura patterns, measure the intensity of each detected mura region, and finally realize automatic defect detection and accurate grading. After the 20th century, more and more industry experts and scholars have developed advanced mura detection algorithm technology based on traditional machine vision algorithms, and continue to explore the research depth of mura inspection. For example, the global image reconstruction scheme based on two-dimensional Fourier transform [6], directional filter group DFB [7], multi-level threshold technology [8], discrete wavelet transform [9], background reconstruction algorithm based on discrete cosine transform DCT [10], and so on. In 2009, Chen et al. first applied neural network [11] method to automatically learn mura defect detection parameters. In the following years, they made many breakthroughs in background separation and reconstruction and defect quantification, and B-spline curve background fitting [12], empirical mode decomposition EMD [13], principal component analysis PCA [14], independent component analysis ICA [15], singular value decomposition SVD [16], region active contour algorithm C-V [17], scalable region fitting RSF model [18], etc. In recent years, with the rapid development of deep learning [19] technology, many researchers began to review the application of deep learning in mura detection, and feature fusion algorithm based on unsupervised learning [20], online classifier and transfer learning OSC-TL method [21], etc.

The main difficulties of mura inspection are as follows: 1. the defect boundary is fuzzy, unable to be clearly separated from the background, and the contrast is weak, so it is difficult to quantify defects; 2. because the overall gray level of the display area is uneven due to the self imaging of optical images, most of the industry currently adopts two methods, namely, improving the defect feature intensity and eliminating background interference. Some researchers will develop customized algorithms for certain mura defect shape contours [22]. Additionally, difficulties in mura defect detection, have impacts on production and manufacturing. For example, mura defects appear as ’blocky uneven brightness,’ with low contrast, irregular shapes, and most defects have no discernible patterns. These observations manifest during the production process, including types like line mura, spot mura, and region mura. Traditional methods classify mura defects based on parameters such as average brightness, space size, area, shape, and center of gravity position [23]. A minor defect in the production process may result in irreparable quality issues for a finished LCD panel.

Due to low contrast and irregular shapes, it makes challenging for industrial production. Automated optical inspection (AOI) technology faces the challenge of sporadically defects occurring during the production process and lead to difficulties in acquiring the substantial amount of defect data and constructing the comprehensive dataset regarding color mura defects [24]. Tradition mura defect detection methods cannot efficiently separate the defect features from the gray background level due to close similarity between backgrounds and color mura defect regions.

For these difficulties of mura defect detection, this paper adopts the method of small sample deep learning to overcome the interference of uneven background. In image preprocessing, it automatically identifies the gray range of the screen display area, and then automatically carries out gray linear stretching to improve the defect feature strength. By adjusting the characteristic amount of the input image, the amount of invalid information in deep learning can be reduced, to improve the efficiency of deep learning. Deep learning methods show limitations in learning rate due to small size dataset and lead to a local optimum. A different activation function can be used to train a network [25]. Moreover, imbalance between normal data and catastrophic data, make deep learning methods to show poor performance. Therefore, unbalanced data feature extraction is a critical issue for the performance of small sample deep learning methods [26]. Different from the traditional way of reducing the uneven interference of the background [27], this paper uses the gradient edge linear stretching method in image preprocessing to enhance the contrast of defects and background at the same time. This data set can automatically identify the background area and defect area through the model architecture of deep learning, which has both efficiency and accuracy. Through the traditional sample enhancement method of machine learning [28], such as rotation, symmetry, scaling, offset, noise, etc., combined with the defect feature automatic fusion algorithm model, the sample set is increased twice to meet the data demand of the project. The experimental results show that the organic light emitting semiconductor detection model based on small sample deep learning proposed in this paper can achieve better detection effect for dot mura defects with weak phenomenon, and the algorithm is simple and efficient. It has strong versatility and is very suitable for the implementation of projects at industrial sites.

Overall the contribution of this paper is as follows:

- This paper proposes the SmartMuraDetectin model based on small sample deep learning to overcome the interference of an uneven background.
- This paper employs the gradient edge linear stretching method for image preprocessing, simultaneously enhancing the contrast of defects and background.
- This paper presents an empirical evaluation of the small sample deep learning method on a dataset, demonstrating better efficiency and accuracy compared to other methods.

## Related studies

A lightweight framework, namely LDS-YOLO, is proposed to extract features from previous layers. The proposed framework retains detailed information about objects and ensures that small targets are not missed. The LDS-YOLO performed better in detecting dead trees and achieved an average precision of 89.11% [29]. Compared to the LDS-YOLO framework, QueryDet utilizes a novel query mechanism to accelerate the inference speed of feature pyramid-based object detectors [30]. This involves locating small objects on low-resolution features before computing accurate detection results on high-resolution features. Prior to these studies, YOLO was developed to detect illegal buildings. Building images often have small targets that vary in size, leading to low detection accuracy. The YOLO-3 algorithm is proposed, incorporating k-means clustering, an optimized loss function, and soft-NMS along with an improved and optimized dataset [31]. Compared to the original YOLO-3, the extended version of the model achieved better detection accuracy.

Deep learning models have applications in computing the diagnosis of myocardial infarction. However, these models can be expensive and time-consuming. To address this issue, adversarial and boundary mining models are proposed. The adversarial learning model complements the boundary mining tool by enhancing its learning from additional unbalanced data [32]. Compared to the state-of-the-art methods, the proposed model demonstrates superior results in myocardial infarction segmentation.

Survival prediction of heart failure (HF) patients is crucial for healthcare management. A motion-based survival prediction method has recently been proposed, capturing the myocardial border [33]. Border features are highly discriminative. A multi-modality deep Cox model predicts the risks of heart failure and aims to improve the survival probability of HF patients. The precision and recall values of the proposed models are 85.19% and 83.33%, respectively. These values can be further enhanced in future work.

For the detection of defects in OLED cells, a finite element method (FEM) has recently been employed to generate virtual dark spot images. Two categories of images are considered: images of dark spots and images obtained after 10,000 hours. Additionally, deep learning models such as CNN, VGG-16, and ResNet-50 are utilized for optimal deep learning [34]. Among these models, VGG-16 exhibited superior performance in detecting dark spot images with a detection accuracy of 98.80%.

A fusion strategy proposed is based on the point-wise mutual information (PMI) and preserves salience features [35]. Moreover, the proposed strategy keeps spatial consistency for a fusion image.

For mura defect feature fusion, a multichannel mechanism is required to process separately the color and luminance features. However, complexity of the color features exceeds the variation range luminance feature [36]. A recent work highlights the comparison of several fusion methods. Among these methods, the proposed “element-wise feature fusion network” EFFN performs better for defect detection for display channels [37]. Based on F1-Measure, and fastest detection speed metric results, the proposed EFFN method received best F1-Measure and speed values. However, the proposed method has not been evaluated in challenging cases to optimize the proposed approach for better accuracy results.

## Theoretical basis

### Feature fusion theory

Small sample target detection usually uses transfer learning combined with sample enhancement to optimize the model training strategy and improve the number of samples. Based on this, according to the characteristics of industrial images, this paper proposes a feature fusion method to fuse defect features on positive samples and generate a large number of negative samples. The main algorithm principle is image fusion.

In the image fusion task, when the foreground is placed on the background, two points need to be guaranteed: the main content of the foreground itself is as smooth as possible compared with the background; the boundary is seamless, that is, the pixel values of the foreground and background at the boundary points need to be consistent. Smoothing can be understood as the same gradient of the image foreground and background, and consistent boundary can be understood as the same pixel value on the boundary. The algorithm idea in this paper is based on the image fusion algorithm in the field of image processing [38]—Poisson Blending. It is different from the traditional rigid fusion method of direct superposition of two images, the core idea of Poisson fusion is to let the target image (dst) grow a new image according to the gradient field of the source image (src) in the fusion part. The fusion image generated in this way not only retains the characteristics of the target image, but also fuses the gradient characteristics of the source image, so the fusion result is very natural, and there is no need for background removal operations such as manual clipping. If the target image is given a fine mask, the perfection of fusion can be further improved [39]. This gradient field guided interpolation operation is also known as "Poisson image editing". The simplest interpolation result is the solution of the minimization problem of the following equation:

$$\begin{array}{c} \underset{f}{min}\;\iint_{\Omega }|\nabla f{|}^{2}with\;{f|}_{\partial \Omega }={f^{*}|}_{\partial \Omega },\\ where\;\nabla .=[\frac{\partial \overset{˙}{\partial }}{\partial x},\frac{\partial \cdot }{\partial y}]\;is\;the\;gradient\;operator. \end{array}$$ (1)

**Fig 2:**
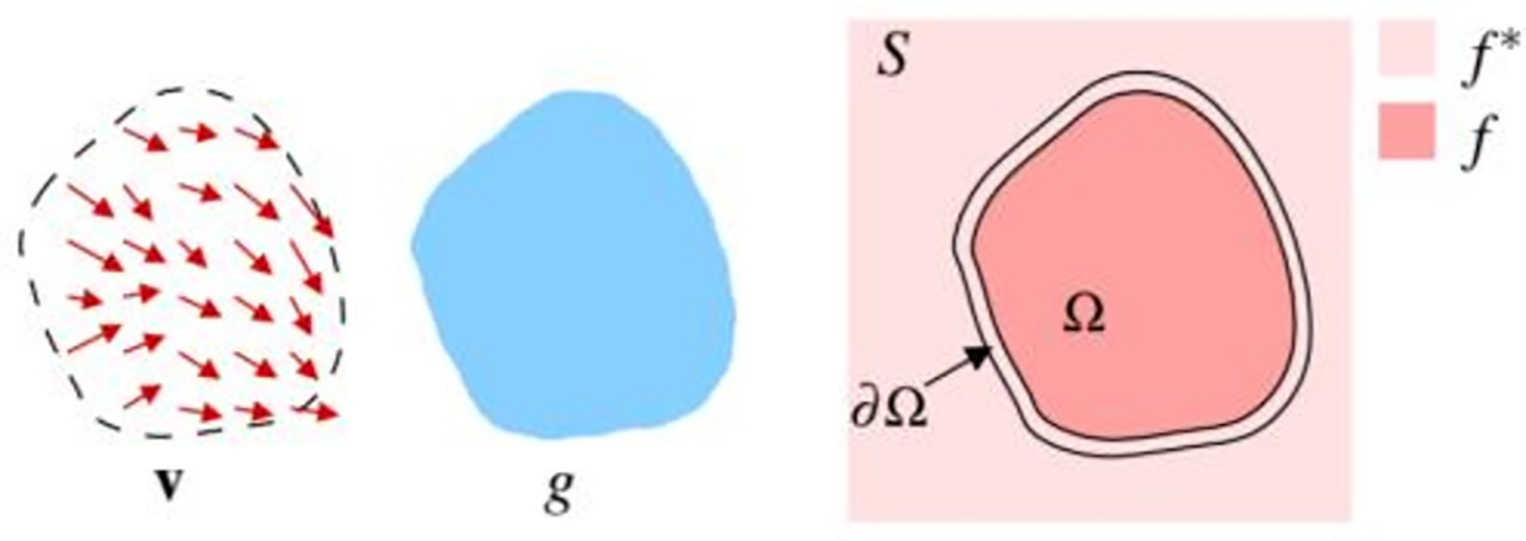
Schematic diagram of the algorithm process of Poisson fusion.

The principle of Poisson fusion algorithm is described in Fig 2 below:

In the above Fig 2, *S* is a closed subset of a two-dimensional real number set ℝ^2^, *Ω* is a closed subset with a boundary of ∂Ω. f* is a function of the set part of *S*−*Ω* (if it is an image, it refers to the gray value of all pixels), *f* is a function of the set *Ω* (that is, a function that needs to be solved by solving the Poisson equation), and *v* is a vector field of the set *Ω* (required for constructing the Poisson equation). According to the different processing of the gradient field (that is, the vector field *v* in Fig 2), Poisson fusion can be divided into normal clone, and mixed clone. There are three categories of monochrome transformer.

### Target detection model

**Fig 3:**
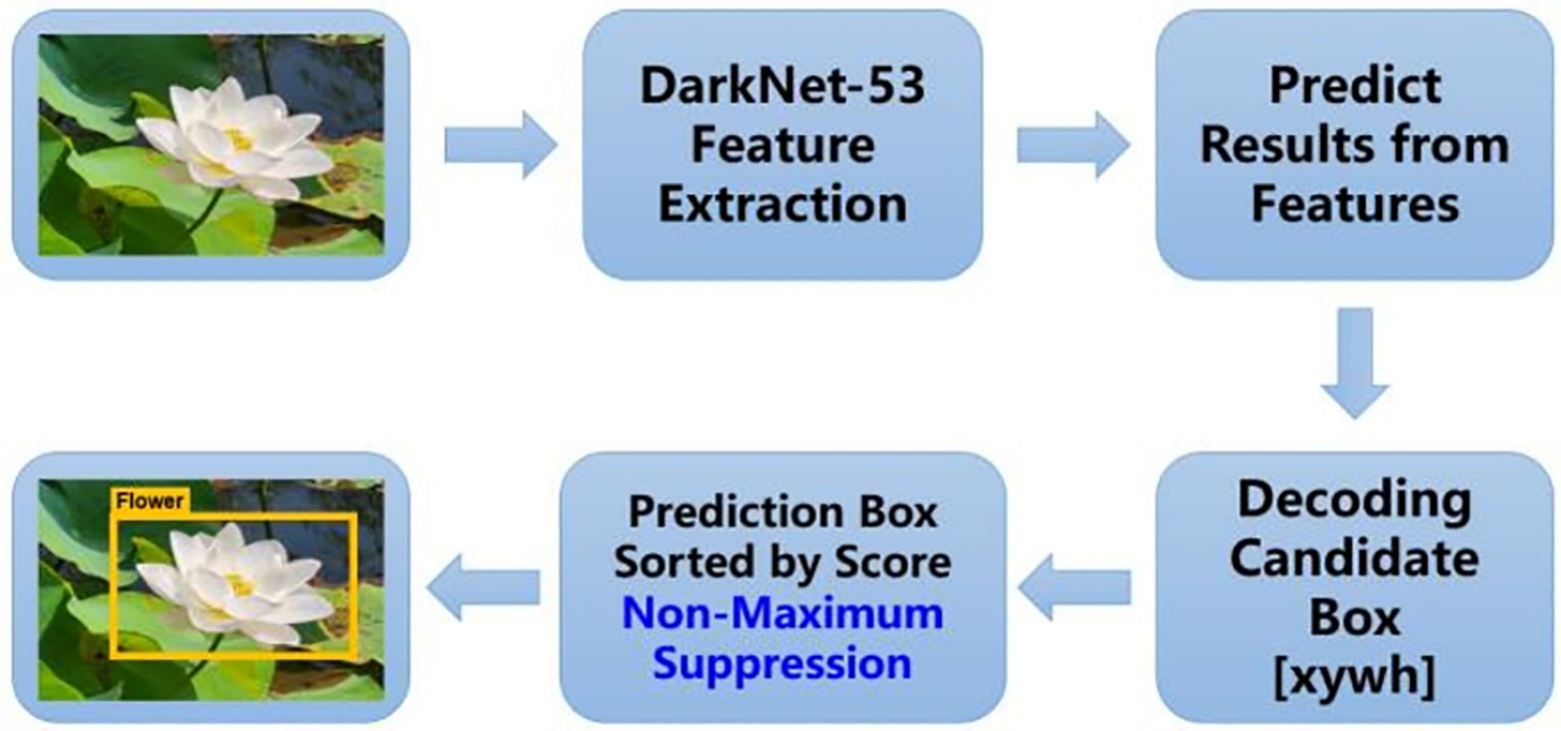
Yolo V3 algorithm flow description.

After preprocessing and enhancement, the original image can be input into the network model of deep learning as sample data. This paper selects the classic Yolo [40] series models in the field of target detection. The Yolo target detection model is a typical One-Stage detection framework, which has obvious speed advantages compared with the previous Two-Stage framework, such as Faster R-CNN [41], and has no limit on the size of the input image. It has excellent engineering practice performance. The algorithm process of Yolo v3 target detection mainly includes feature extraction, result prediction, decoding candidate box, score sorting, non-maximum suppression and optimal target acquisition, as shown in Fig 3.

In this paper, the Yolo v3 model, which is commonly used in landing projects in the industrial field [42], is used. The accuracy performance of Yolo v3 reasoning is compared with other target detection models as shown in Table 1.

**Table 1:** Performance comparison between YOLO v3 and other target detection networks.

Two-stage methods	Backbone	AP	AP_50_	AP_75_
Faster R-CNN+++	ResNet-101-C4	34.9	55.7	37.4
Faster R-CNN w FPN	ResNet-101-FPN	36.2	59.1	39.0
Faster R-CNN by G-RMI	Inception-ResNet-v2	34.7	55.5	36.7
Faster R-CNN w TDM	Inception-ResNet-v2-TDM	36.8	57.7	39.2
**One-stage methods**				
YOLOv2	DarkNet-19	21.6	44.0	19.2
SSD513	ResNet-101-SSD	31.2	50.4	33.3
DSSD513	ResNet-101-DSSD	33.2	53.3	35.2
RetinaNet	ResNet-101-FPN	39.1	59.1	42.3
RetinaNet	ResNeXt-101-FPN	40.8	61.1	44.1
YOLOv3 608×608	DarkNet-53	33.0	57.9	34.4

**Fig 4:**
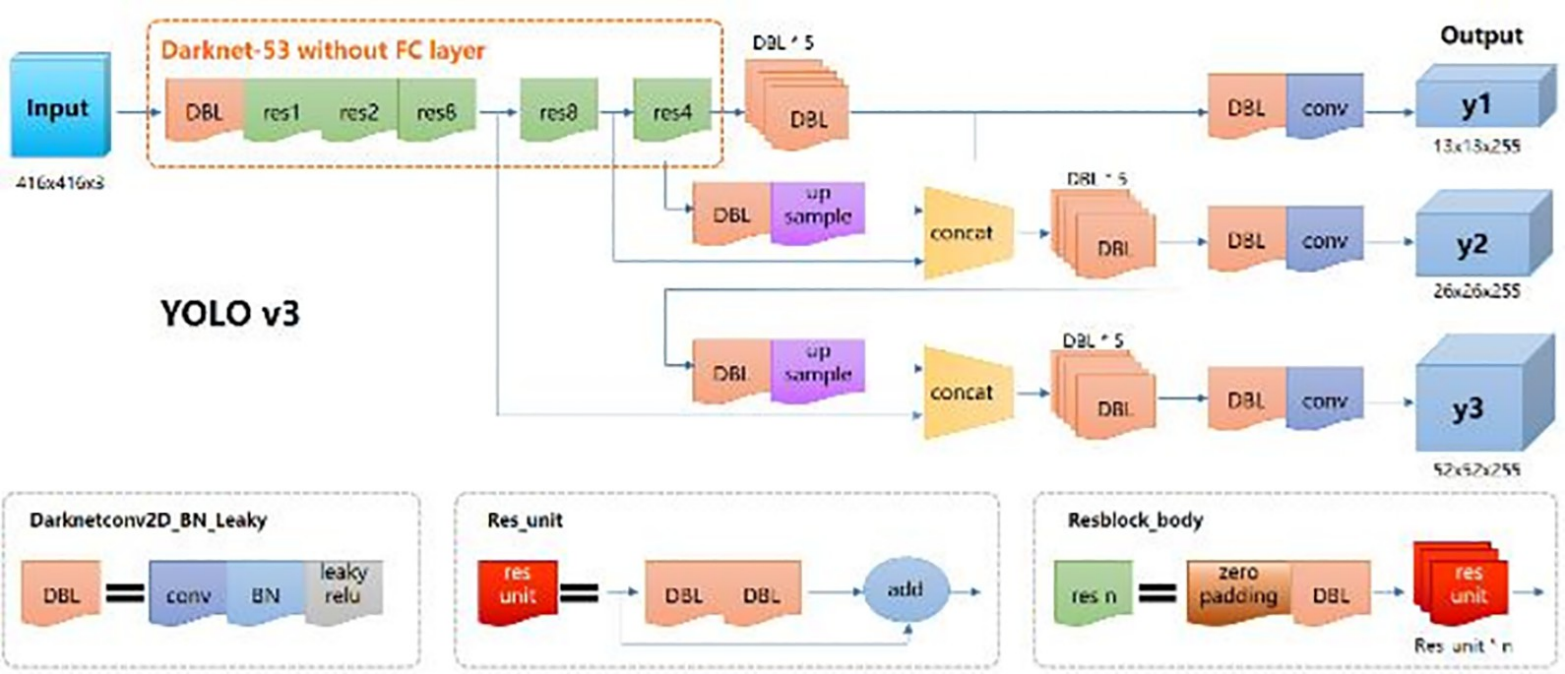
Network structure of Yolo v3.

The network structure of Yolo v3 is shown in Fig 4 below:

In the backbone configuration, darknet-53 [43] is selected, and the training strategy adopts the mode of initial warming combined with later cosine delay. At the same time, this paper applies k-means algorithm to cluster 9 groups of width and height data on mura labeled dataset for 9 candidate box anchors of 3 scales, which makes the regression performance of the search box inferred by the model more accurate.

### Detection model of organic light emitting diodes based on small sample deep learning

**Fig 5:**
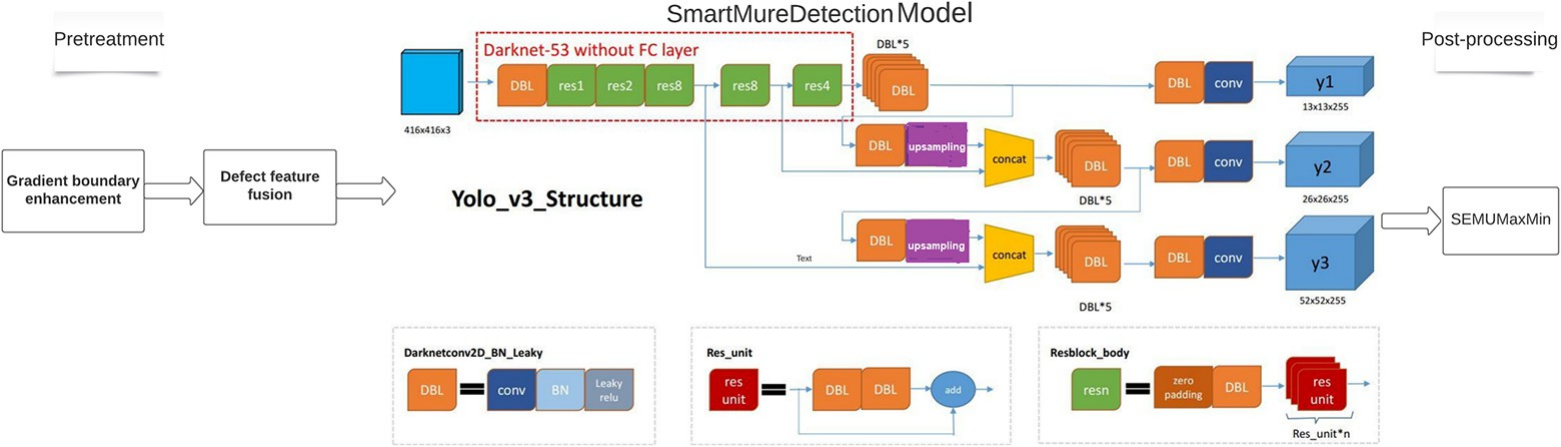
SmartMuraDetection model structure.

Aiming at the four main problems of weak defect characteristics, insufficient negative sample size, small defect scale and difficult to quantify the output characteristics, combined with the gradient boundary enhancement algorithm module, defect feature fusion module, small-scale target detection TinyDetection model and SEMUMaxMin quantization module, this paper constructs the organic light emitting diodes detection model SmartMuraDetection based on small sample deep learning, and the model architecture is shown in Fig 5 below.

### Gradient boundary enhancement algorithm module

**Fig 6:**
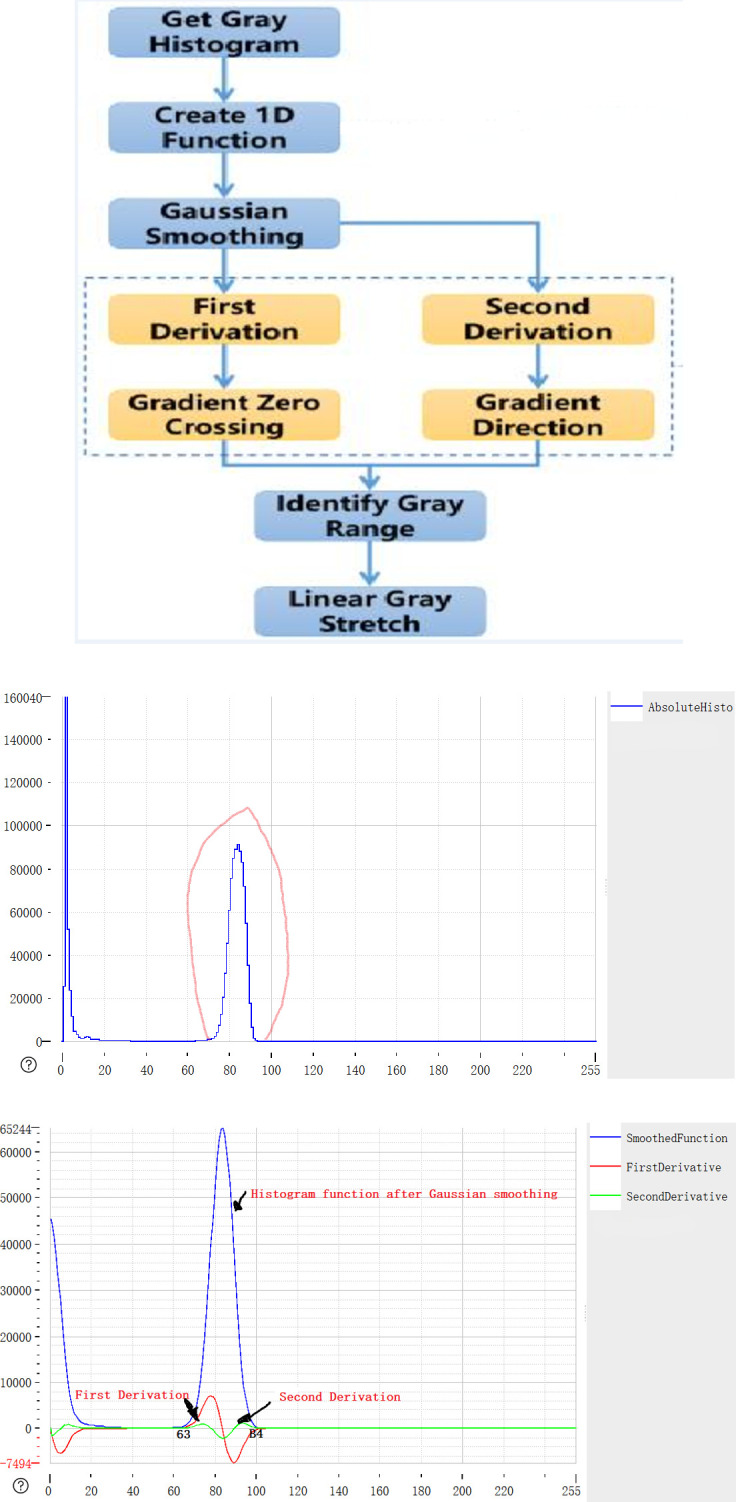
Process of feature enhancement algorithm.

Gradient boundary enhancement algorithm mainly solves the problem of weak defect features, enhances the effective contrast of the image, and takes into account the maximization of defect features and no loss of effective information [44]. The main principle of feature enhancement is to find the gray range of the display area through the second-order derivation of the image, and then carry out automatic linear gray mapping to achieve the effect of contrast amplification. The algorithm flow and related diagrams are depicted in Fig 6(A), while Fig 6(B) and 6(C) display specific details.

The main idea is to automatically find the high-order gray-scale region with a large area of pixel accumulation on the histogram. First, this paper obtains the gray-scale histogram of the image. From the histogram, it can be seen that the main distribution range of pixels is about 0 ~ 10 background dark field and about 60 ~ 100 screen display bright field. This paper needs to extract the gray-scale range of the bright field region. After obtaining the histogram, a one-dimensional function is created to facilitate the subsequent function operation. The one-dimensional function is smoothed by Gaussian smoothing [45] to eliminate interference and obtain the smoothed function. Then, the first-order derivative and the second-order derivative of the smoothed function are obtained, in which the first-order derivative reflects the gradient change of the function, and the second-order derivative reflects the trend direction (concave convex) of the gradient change of the function. By obtaining the zero crossing point of the first-order derivative, the extreme points of the function can be obtained. Combined with the directionality of the second-order derivative, finally, the gray scale range of the highlighted area of the histogram can be automatically obtained. In this example, the upper and lower limits of the gray scale of the display area automatically obtained are 63 and 84, which accurately reflect the bright field gray scale range of the screen display area.

After automatically obtaining the gray range of the highlighted area, through pixel gray mapping, this paper can linearly stretch the image gray to achieve the effect of contrast enhancement:

$$D_{B}=f(D_{A})=aD_{A}+b$$ (2)

**Fig 7:**
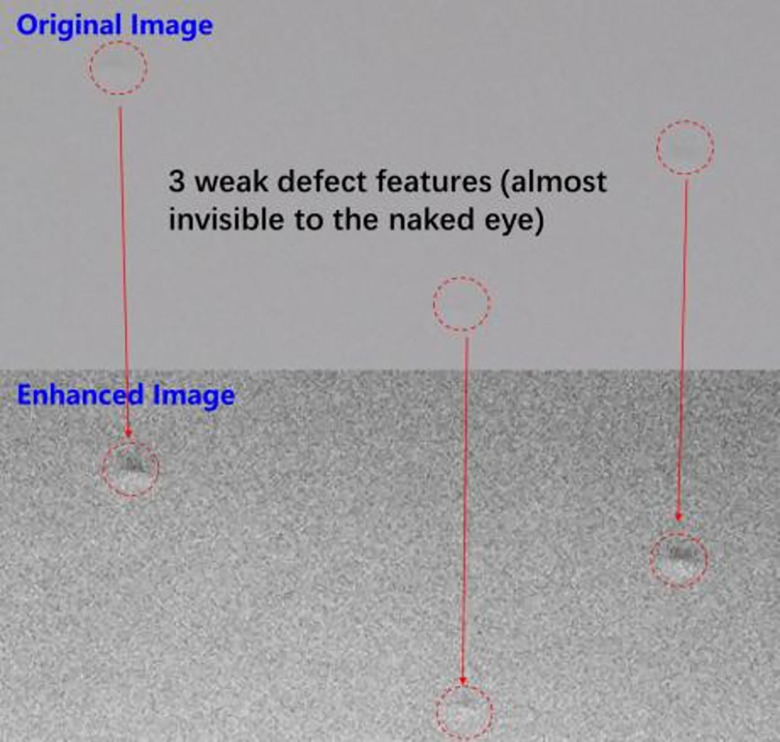
Defect feature enhancement effect.

Compared with the traditional direct contrast enhancement algorithm, this feature enhancement algorithm increases the accuracy of the enhanced gray range, which can maximize the enhancement of defect features. The operation effect of the algorithm is shown in Fig 7. At the same time, due to automatic coding feature of deep learning, this algorithm does not need to consider the uneven gray level of the reconstructed background. It can be automatically recognized by subsequent deep learning target detection. Because there is no need for background separation or background reconstruction, the simplicity and robustness of the algorithm are greatly improved.

### Defect feature fusion module

The defect feature fusion module mainly solves the problem of insufficient negative samples by embedding a defect region in the source image into the target image to generate a new negative sample image and solve the problem of insufficient negative samples and unbalanced samples. In this paper, the method of constructing Poisson equation to solve the optimal value of pixels is used to fuse the source image and the target image while preserving the gradient information of the source image. There are three main fusion methods: (1) NORMAL_CLONE, which does not retain the texture details of the target image, and the gradient of the target area is only determined by the source image; (2) MIXED_CLONE, which retains the texture details of the target image, and the gradient of the target area is calculated by the combination of the source image and the target image; (3) MONOCHROME_TRANSFER, which does not retain the color details of the source image, only the texture of the target image. The color is the same as the target image. This method can be used to fill the skin texture.

**Fig 8:**
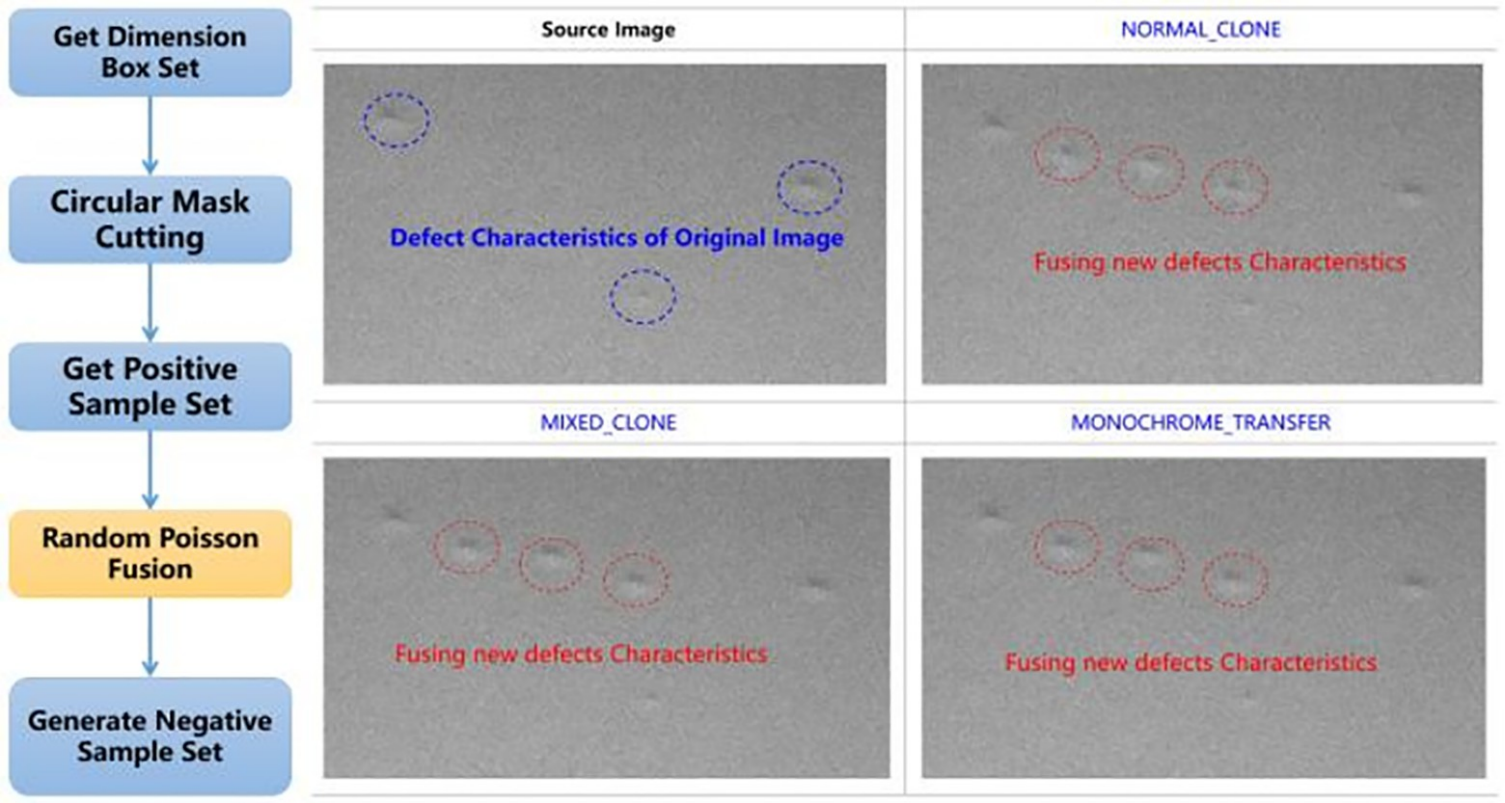
Automatic defect fusion mechanism.

Using the precise fusion of defects requires the production of mask and the setting of object placement area in the target image, which is a cumbersome process. In order to realize the automation of defect enhancement, this paper designs a set of automatic fusion mechanism of defects, as shown in Fig 8.

Firstly, 200 dimension boxes are randomly selected from the marked data set, and 200 circular masks are generated after reducing by 20% according to the size and center of the dimension boxes. Then, small defect images are cut out from the 200 dimension boxes and masks, and 1–10 small defect images are randomly taken out each time after the sequence is disrupted, and are placed at random positions in the screen display area of the positive sample image by Poisson fusion algorithm. According to the size of the randomly placed area of defects in the positive sample and the defect small graph, the annotation information of the data set is automatically generated, and the whole process automation mechanism is realized without manual intervention.

Through a large number of fusion tests of different defects and different backgrounds, combined with the gray contrast of edge transition pixels and the recognition degree judgment of human eyes (on-site certified panel test operator), the new fusion sample obtained by MIXED_CLONE method is the best. Therefore, MIXED_CLONE is selected in this paper. Through the defect feature fusion mechanism, the number of the overall deep learning sample set increases to 2.5 times of the original.

### TinyDetection model for small scale target detection

**Fig 9:**
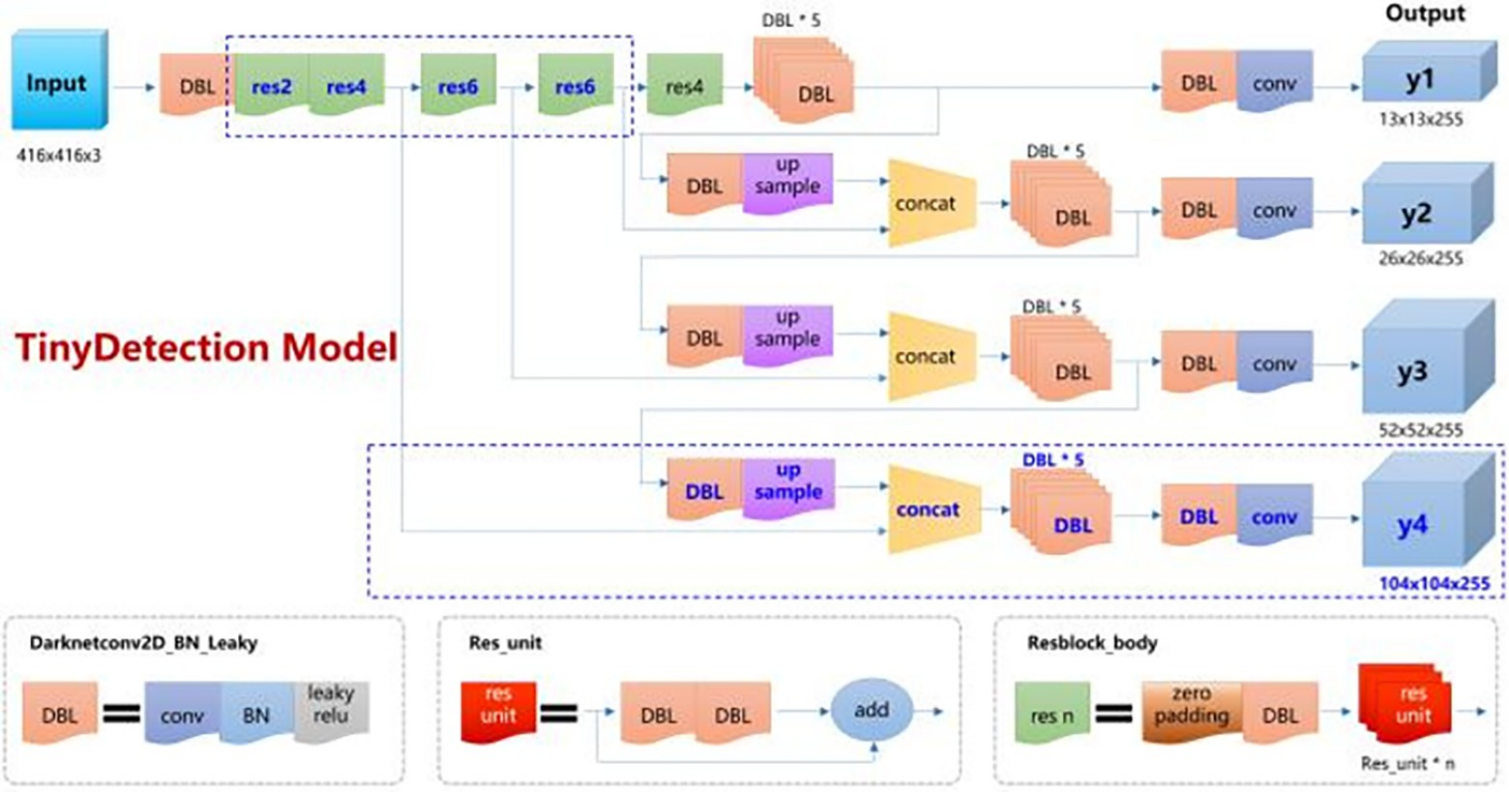
TinyDetection model structure for small scale target detection.

The original input image resolution is 1992 pixel × 601 pixel, defect size is about 15 pixel × 15 pixel, the proportion of defect size in the whole image is very small. In order to accurately detect defects, in addition to pre clustering 9 groups of width and height data on the labeled data set using K-means algorithm, this paper proposes TinyDetection model for small-scale target detection. On the basis of Yolo v3 network, the model increases the residual blocks of shallow network and appropriately reduces the residual blocks of deep network, so that the network can extract the details of small targets more fully. At the same time, the generalization of the model is improved. The number of residual blocks in the first five layers of the original Yolo v3 model is 1,2,8,8,4, respectively, and the number of residual blocks in the first five layers of the optimized model is 2,4,6,6,4, respectively. The new network layer improves the recognition ability of small target details. Then, this paper adds an output feature map with the size of 104 × 104 × 255, which is used for accurate recognition of small targets. Its link mode refers to the original output feature map, which is output from the second residual block and fused with the features of the previous scale. In this way, the features stitched out are richer in target details. The TinyDetection model structure of small-scale target detection is shown in Fig 9. TinyDetection model can significantly improve the accuracy of small target detection while keeping the detection speed unchanged, which is more suitable for this Mura detection task.

### SEMUMaxMin quantization module

After mura defects are extracted through the target detection model, it is necessary to quantitatively evaluate mura, output mura grade index, and combine the threshold to filter the final mura and determine the total results. The international society for semiconductor equipment and materials (SEMI) has established and published a set of measurement and evaluation standards for mura of flat panel displays (SEMU), and proposed the evaluation method of mura defect grade [45].

Before the SEMU value is calculated, the contrast of mura defect under JND (Just Noticeable Difference) shall be calculated first, and the formula is as follows:

$$C_{jnd}=(\frac{1.97}{S_{x}^{0.33}}+0.72)$$ (3)

Where, *C*_*jnd*_ refers to the contrast under JND, and the unit is %; *S*_*x*_ is the area of Mura defect under JND contrast, unit is mm^2^.

The quantification of mura, that is, SEMU value [46], is defined as:

$$SEMU=\frac{\left|C_{x}\right|}{C_{jnd}}=\frac{\left|C_{x}\right|}{\left(\frac{1.97}{S_{x}^{0.33}}+0.72\right)}$$ (4)

Where, *C*_*x*_ refers to the average contrast of mura area, and the unit is %.

The defects inferred from the deep learning target detection model [47] are passed to mura quantitative algorithm for SEMU calculation. If the mura defect judgment level exceeds the set threshold range, the defect is determined to be a real defect, otherwise it is determined to be a non-real defect for good treatment.

**Fig 10:**
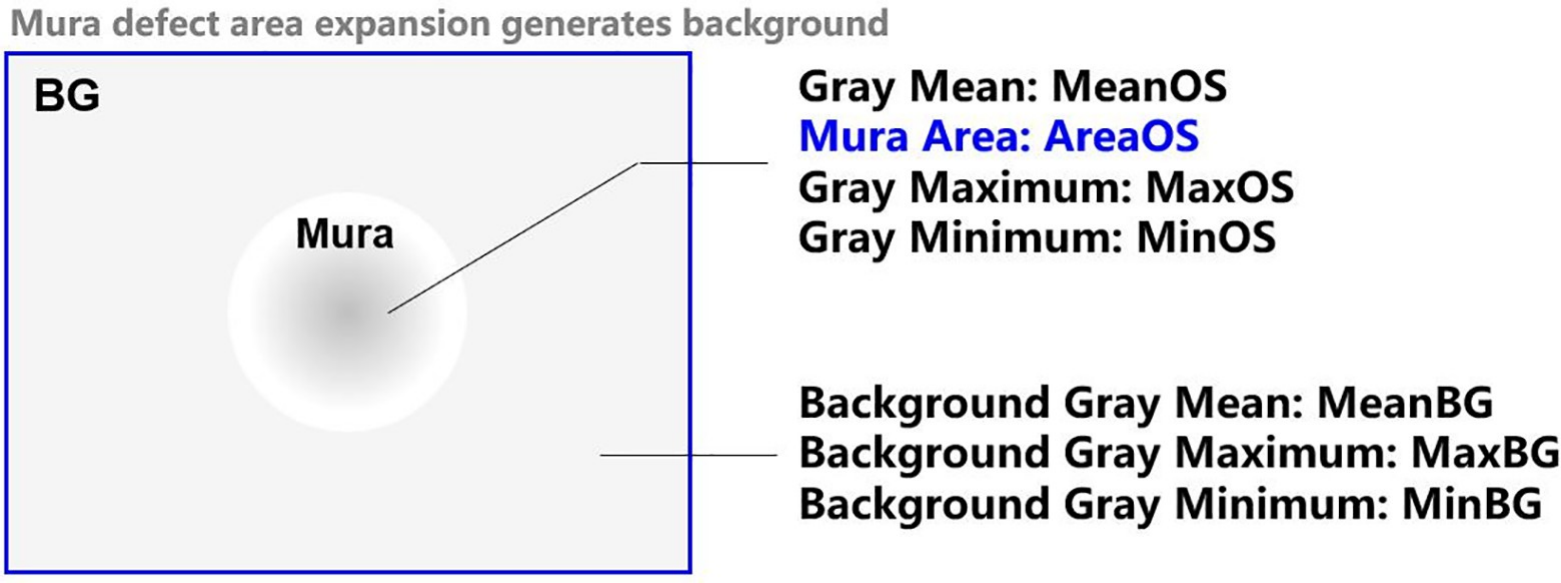
Calculation of Semu value and semumaxmin.

In this paper, the SEMUMaxMin value calculation method is designed. Firstly, the contrast and MaxMin value(the maximum and minimum gray value of the area) of the defect area and the background area are counted, and then imported into the SEMU and SEMUMaxMin calculation formula to get the final result. Fig 10 shows the schematic of SEMU and SEMUMaxMin calculation, in which Mura defect area expands outward to generate the background area. Generally, the expansion amount is set to be twice the length and width of the minimum positive circumscribed rectangle of the defect area, In Fig 10, the mean gray value MeanOS, the area AreaOS, the maximum gray value MaxOS and the minimum gray value MinOS of the defect area are described, and the mean background gray value MeanBG, the maximum background gray value MaxBG and the minimum background gray value MinBG are described.

Step 1: calculate the minimum perceptible contrast *C*_*jnd*_ value of the human eye [48]:

$$C_{jnd}=(\frac{1.97}{{AreaOS}^{0.33}}+0.72)$$ (5)

Step 2: calculate the regional Contrast and ContrastMaxMin:

$$Contrast=\frac{\left|MeanOS-MeanBG\right|}{MeanBG}$$ (6)

$$ContrastMaxMin=\frac{\left|MaxOS-MinOS\right|}{\left|MaxBG-MinBG\right|}$$ (7)

Step 3: calculate SEMU and SEMUMaxMin:

$$SEMU=\frac{Contrast}{C_{jnd}}$$ (8)

$$SEMUMaxMin=\frac{ContrastMaxMin}{C_{jnd}}$$ (9)

## Experiment and result analysis

### Experimental environment and algorithm evaluation index

Based on the detection principle of machine vision, a fully automatic mura detection device is designed and developed in this paper. The visual part of the device is mainly composed of image acquisition module, motion control module, screen drive module and computer operation unit. The supporting external devices include feeding and discharging device, alignment guide device and automatic cleaning device. After obtaining a complete and clear OLED screen image, the input image is sent to a high-performance computer for automatic operation. It is mainly divided into two stages: model training and model deployment reasoning. The computer running environment is CPU Intel Core i7-11700kf, with a dominant frequency of 3.6 GHz 16 cores, a memory size of 64g, a NVIDIA geforce RTX 3090 graphics card with a memory size of 24GB, and the operating system is Window10 21h1. In the model training stage, this paper uses image preprocessing and defect feature enhancement to enhance the original image, then, through the deep learning data enhancement and image defect fusion algorithm, the sample increment is automatically carried out, and the data set with enhanced sample number is put into the deep learning TinyDetection model for model training. In the model deployment reasoning stage, this paper exports the model that has passed the test of the test set, develops the corresponding interface, and deploys it on the detection equipment to reason the images collected in real time on the production line. The mura defect is quantified and filtered by SEMUMaxMin, and the detection result of the corresponding product is finally output to control the subsequent logistics action of the discharging manipulator.

**Fig 11:**
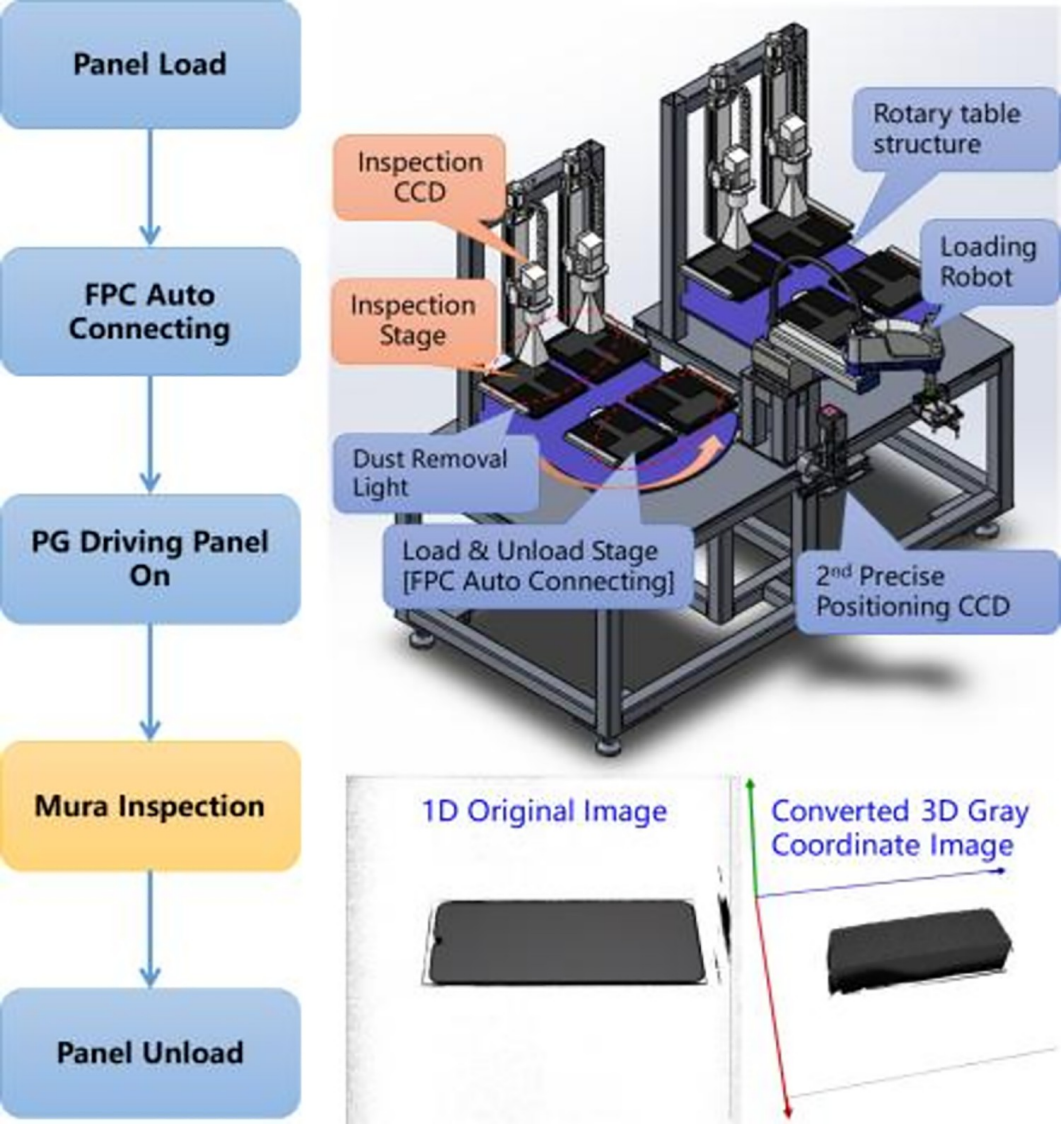
Experimental environment, detection process and drawing effect.

The OLED mobile screen used in this algorithm experiment has a screen length and width of 69.522 mm * 154.56 mm, a screen resolution of 1080 pixel * 2400 pixel, and a German AVT Manta G-609C color industrial camera with a camera resolution of 6 million pixels. This industrial camera uses Sony’s ICX694 CCD global exposure chip. The CCD sensor uses the industry-leading ExView HAD II four channel sensor technology to improve the low light effect and dynamic range. With the characteristics of high sensitivity and low noise, it can meet the rigorous and accurate imaging requirements of OLED image quality inspection. The overall process, device and image acquisition effect are shown in Fig 11.

In terms of the evaluation index of the model, because this experiment tests the target detection performance of a single defect, this paper mainly examines the comprehensive AP value of the network model. The AP value refers to the average precision of a single class. This index includes two indicators: the comprehensive accuracy precision and the recall rate. The accuracy represents the correctness of the box found by the detection box, which measures the degree of false detection. Recall rate refers to the ratio of the correct box found by the monitoring network to the ground truth, which measures the degree of missed detection. AP value represents the average accuracy rate of the detection network in each recall case, and the corresponding area formula under the PR curve is as follows:

$$AP=\frac{\sum P_{r_{i}}}{\sum r}$$ (10)

Where $P_{r_{i}}$ is the p value corresponding to r − i on the PR curve, and ∑r = 1.

Therefore, in the evaluation index of the target detection model, the accuracy of the model can be comprehensively evaluated by comparing the AP value.

### Organic light emitting semiconductor detection experiment based on small sample deep learning

The OLED mobile phone screen image collected by the industrial camera is transformed into a resolution of 1992 pixel × 601 pixel after image preprocessing and defect feature enhancement. The total number of large image data sets collected in this paper is 334pcs, and each large image contains 1 ~ n defects. The image effect of size 416 × 416 is better, and the original large-scale gray image is directly down sampled and compressed to 416 × 416 will lose a lot of details, resulting in the size and area of the defect will be too small, affecting the target detection. So this paper uses the original gray image cutting method to deal with. Considering that the defect may exist at the cutting boundary, about 25% overlap is used to overlap the transition region, so that the size of the original gray image is 1993 × 603 is divided into width and height 6 × 2 total 12 sheets size 416 × 416, and the annotation position of the corresponding thumbnail is automatically converted and generated. The total number of thumbnail data sets after automatic cutting is 4008pcs. In order to quickly cut the image and reduce the manual re-annotation load, this paper develops an automatic image segmentation tool, which loads the original data set and original annotation information in batches, automatically identifies the image edge and segmentation span, preprocesses all data sets at one time, and obtains the input data set of the model.

**Fig 12:**
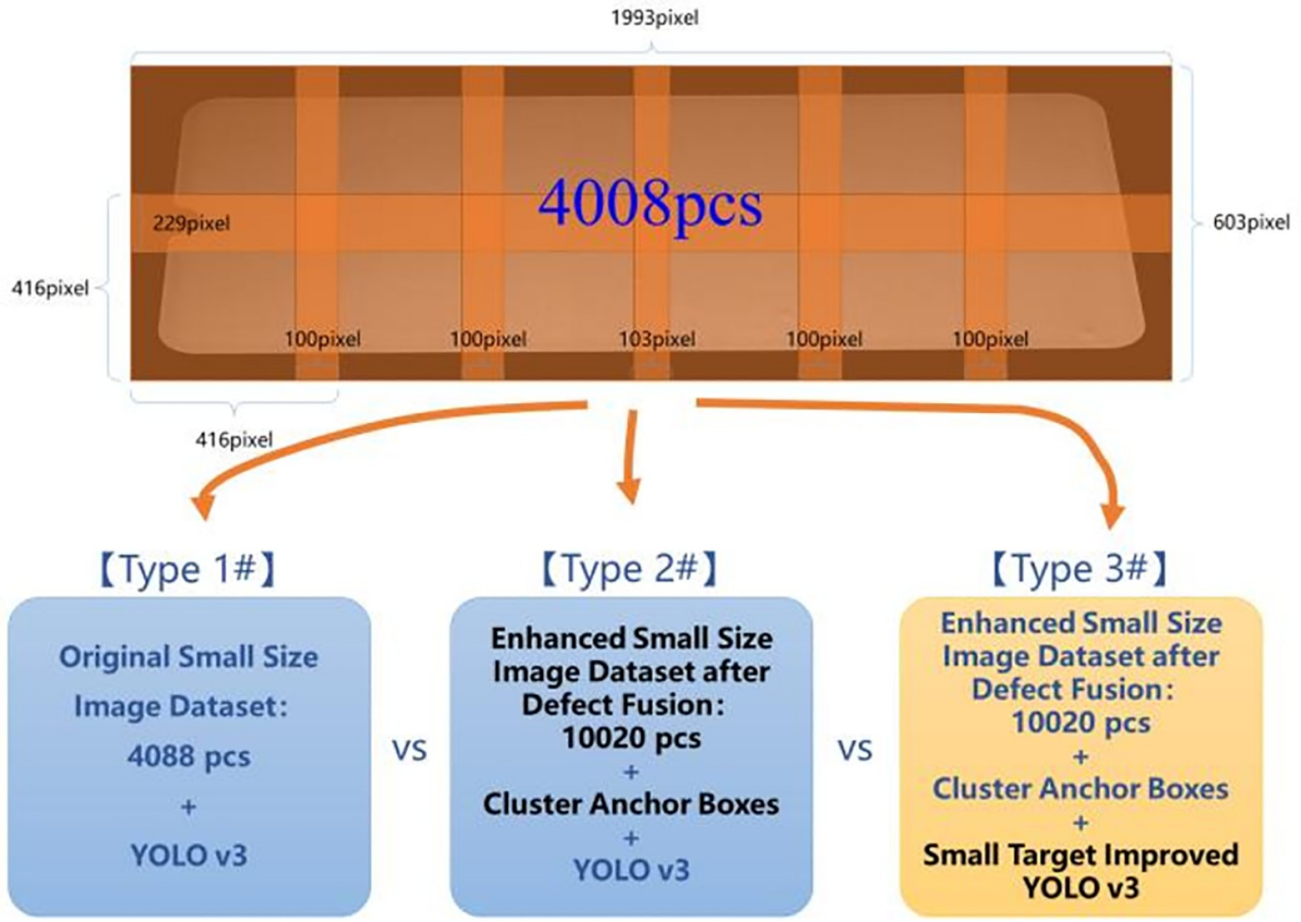
The difference of three kings of deep learning target detection.

After obtaining the training set, this paper uses the iterative optimization method to test the performance differences of different algorithms. In experiment 1, the automatically trimmed small graph training set is directly input into the Yolo v3 model for training and evaluation. In Experiment 2, defect fusion was performed on the data by increasing the number of data sets and inputting them into the Yolo v3 model. The pre-selected box size was generated by clustering [49] as a super parameter to train and evaluate the target detection model. On the basis of experiment 2, experiment 3 customized and optimized the Yolo v3 network model according to the characteristics of small target detection [50], and input the incremental data set obtained in mode 2 into the TinyDetection model of small-scale target detection to test its training and evaluation performance. Pytorch was used in the deep learning framework, and the comparison of data sets and three experimental schemes is shown in Fig 12:

On the superparameter setting of the network model, the number of training rounds epochs is 300, and the learning rate is set to 1e-3, the momentum parameter is set to 0.9, the weight attenuation canonical decay is set to 0.0005, batch size is set to 64, iou_thres is set to 0.5, conf_thres is set to 0.4, nms_thres is set to 0.4, and the input image data set is normalized. The initialization weight of the model is the pre training weight on the official VOC data set.

The data set before enhancement is input to the Yolo v3 network model in the experiment 1. The number of image data sets is 4008 pcs, including 1964 pcs of good product images and 2044 pcs of defective images. The total number of marked defective area boxes is 2722 ea.

In Experiment 2, the data set was enhanced by the defect fusion algorithm. The number of enhanced image data set was 10020 pcs, including 5608 pcs of good image, 4412 pcs of defective image, and the total number of labeled defect area boxes was 5791 ea. In order to better adapt to the defect characteristics of the data set in the industrial scene, this paper used k-means clustering to calculate the labeled data, 9 groups of anchor box sizes are obtained. Different from the characteristics of large size differences and different shapes of image datasets in the Internet and consumer fields, defects in the industrial field are generally of single type, and the size and shape are relatively similar and stable. Therefore, the pre-selection box is obtained by clustering, which can more accurately match the actual mass production image defect scene. Through the use of k-means algorithm ideas, combined with iou to replace the traditional Euclidean distance to measure the deviation, and continuous iterative updates, 9 groups of data are finally obtained. The pre selection box values obtained from the data set in this experiment are [14.27, 12.52], [20.79, 16.11], [26.81, 17.26], [26.17, 21.58], [26.52, 26.87], [33.17, 20.09], [32.29, 25.31], [39.00, 26.51], [46.19, 33.52], and the loss of k-means clustering process is 771.90. The pre selection boxes above have been sorted according to three sampling scales of small, medium and large, and written into the configuration of Yolo v3 for application.

In experiment 3, on the basis of experiment 2, the model was replaced by TinyDetection model for small-scale target detection, and trained on the data set. Because the new model has no mature pre-training weight, this paper used VOC data set for pre-training of the model on multi card GPU cluster server, so that the model has the preliminary ability of image feature extraction, and then loaded into the local data set for re-training of the model TinyDetection model for small-scale target detection is mainly optimized for small-scale target detection to improve the generalization of the model, as well as the accuracy of small-scale target detection and prediction frame accuracy.

**Fig 13:**
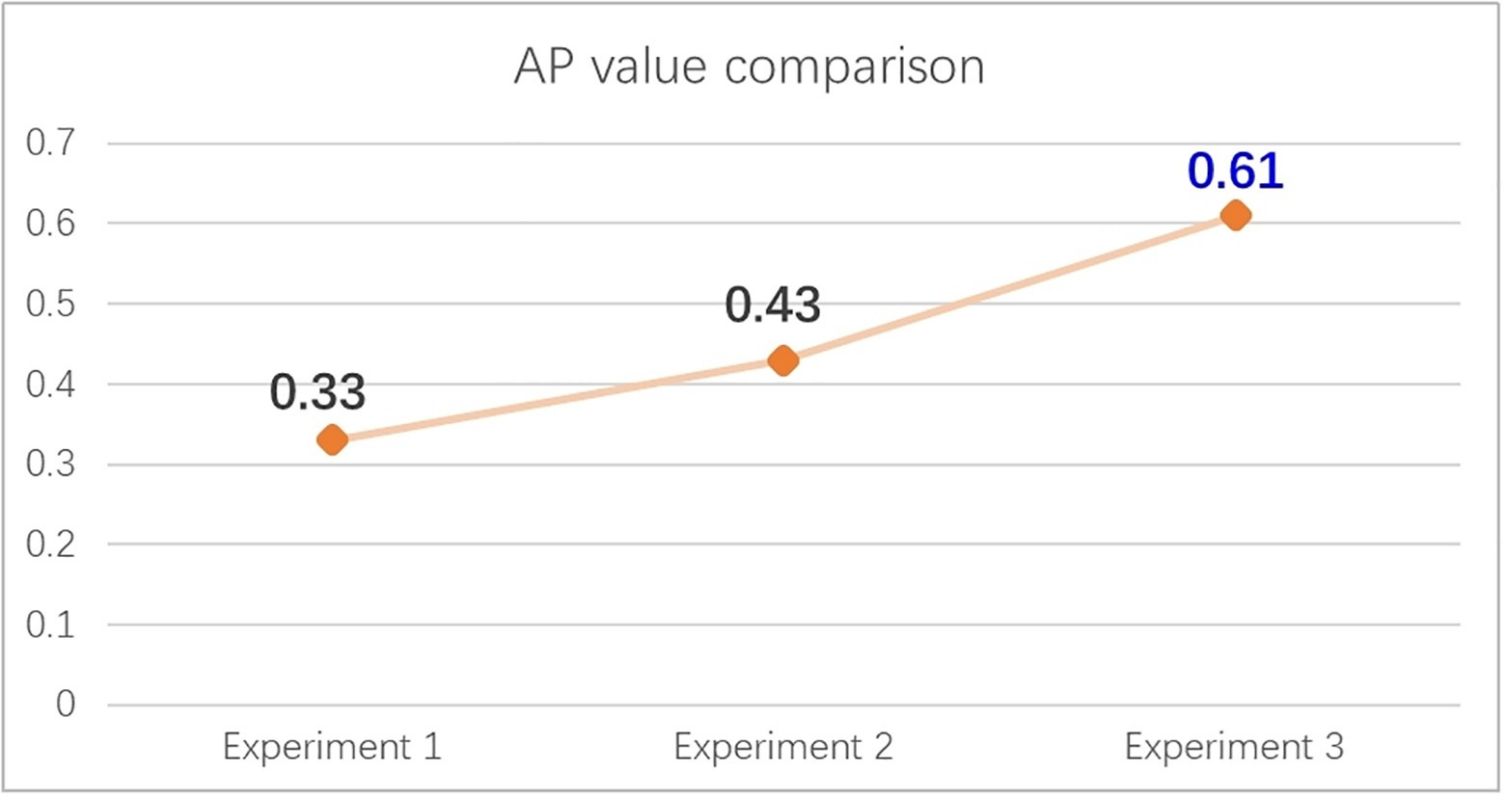
Comparison of AP values of three experiments.

**Fig 14:**
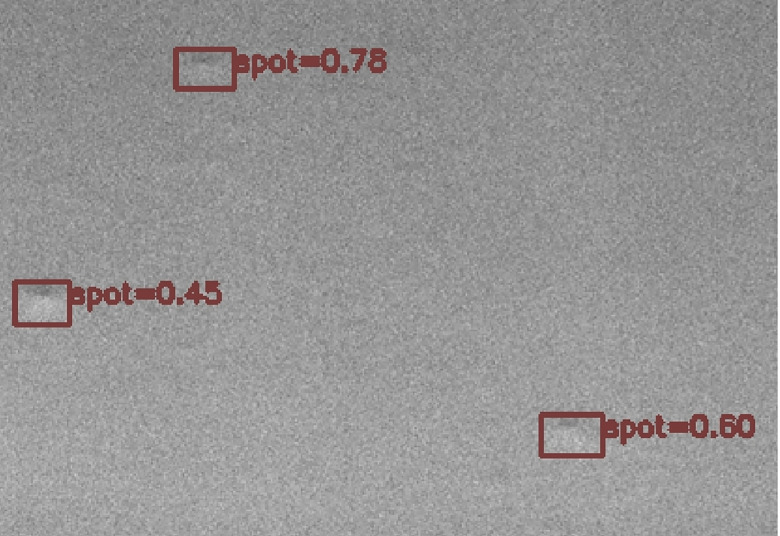
Results of TinyDetection model.

In experiment 1, the AP value is 0.33, and the average accuracy has reached the elementary level. The AP value of experiment 2 was 0.43, which was higher than that of experiment 1 on average and achieved better inspection effect. The data set enhanced by defect fusion algorithm obtained better accuracy and had obvious detection advantages; In experiment 3, the AP value was 0.61, and the TinyDetection model for small-scale target detection got better accuracy for small target detection, which had the best detection performance in the three model training experiments. The experimental comparison results are shown in Fig 13:

The best model weight after training is saved locally. After reloading the model network and weight, this paper randomly extracts an image from the test set for reasoning test, and obtains the result image of the identification prediction boxes, as shown in Fig 14.

**Fig 15:**
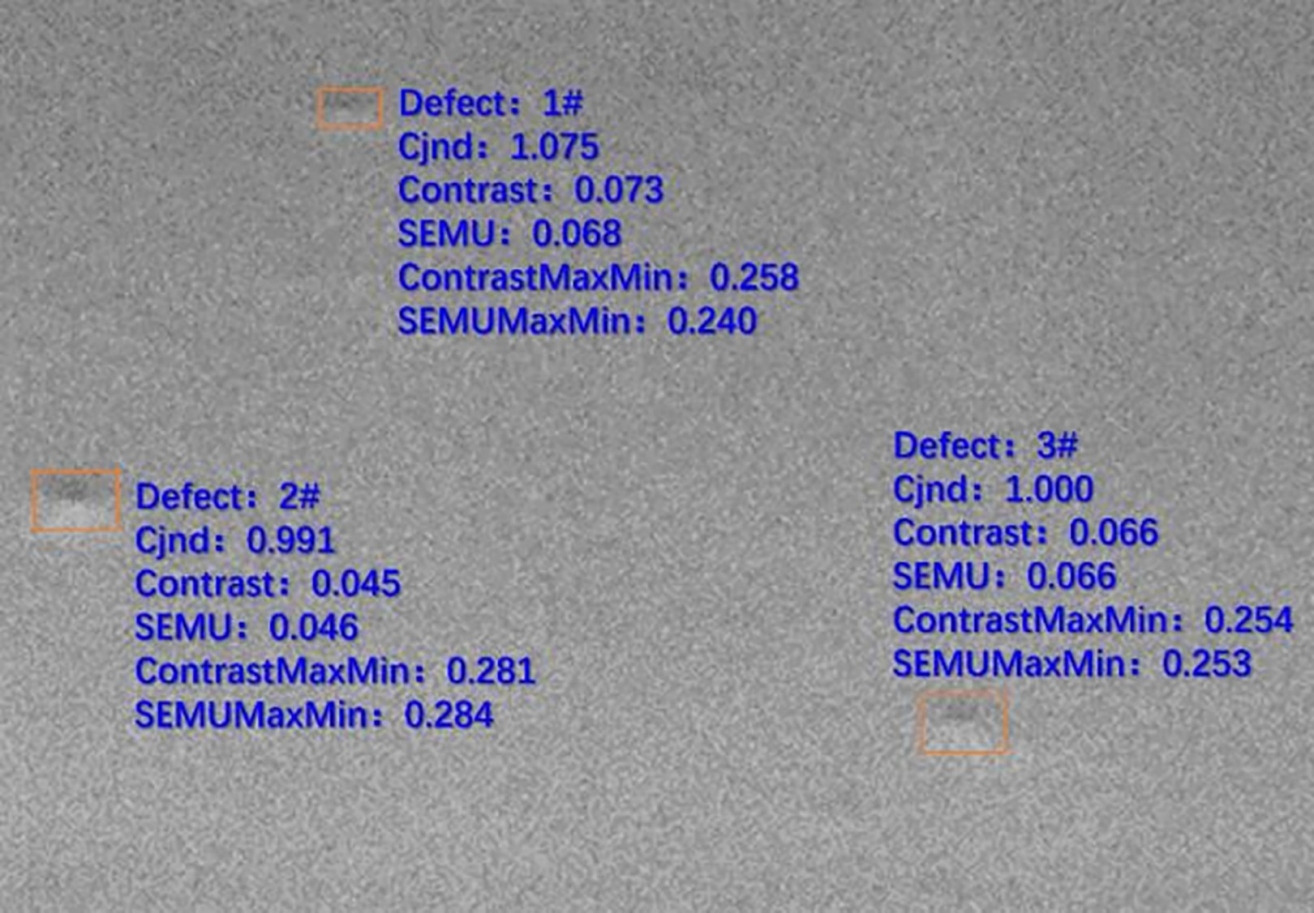
Calculated contrast and SEMUMaxMin result.

For the result image of annotation prediction boxes obtained in the previous model reasoning link, this paper uses SEMUMaxMin calculation method to accurately quantify the identified defect area and obtain the results of each defect intensity value of the image. The calculated contrast and SEMU value are shown in Fig 15.

According to the obtained result data, as shown in Table 2, the SEMUMaxMin algorithm independently developed in this paper better matches the degree of human eye inspection than the traditional SEMU algorithm [51]. In this example, it is sorted according to the degree of defect, that is, defect 2 is the most obvious, defect 3 is the second, and defect 1 is the weakest.

**Table 2:** Calculated contrast and Semu results.

Defect Number	Cjnd	Contrast	Semu	Contractmaxmin	Semumaxmin
**1#**	1.075	0.073	**0.068**	0.258	**0.240**
**2#**	0.991	0.045	**0.046**	0.281	**0.284**
**3#**	1.000	0.066	**0.066**	0.254	**0.253**

**Fig 16:**
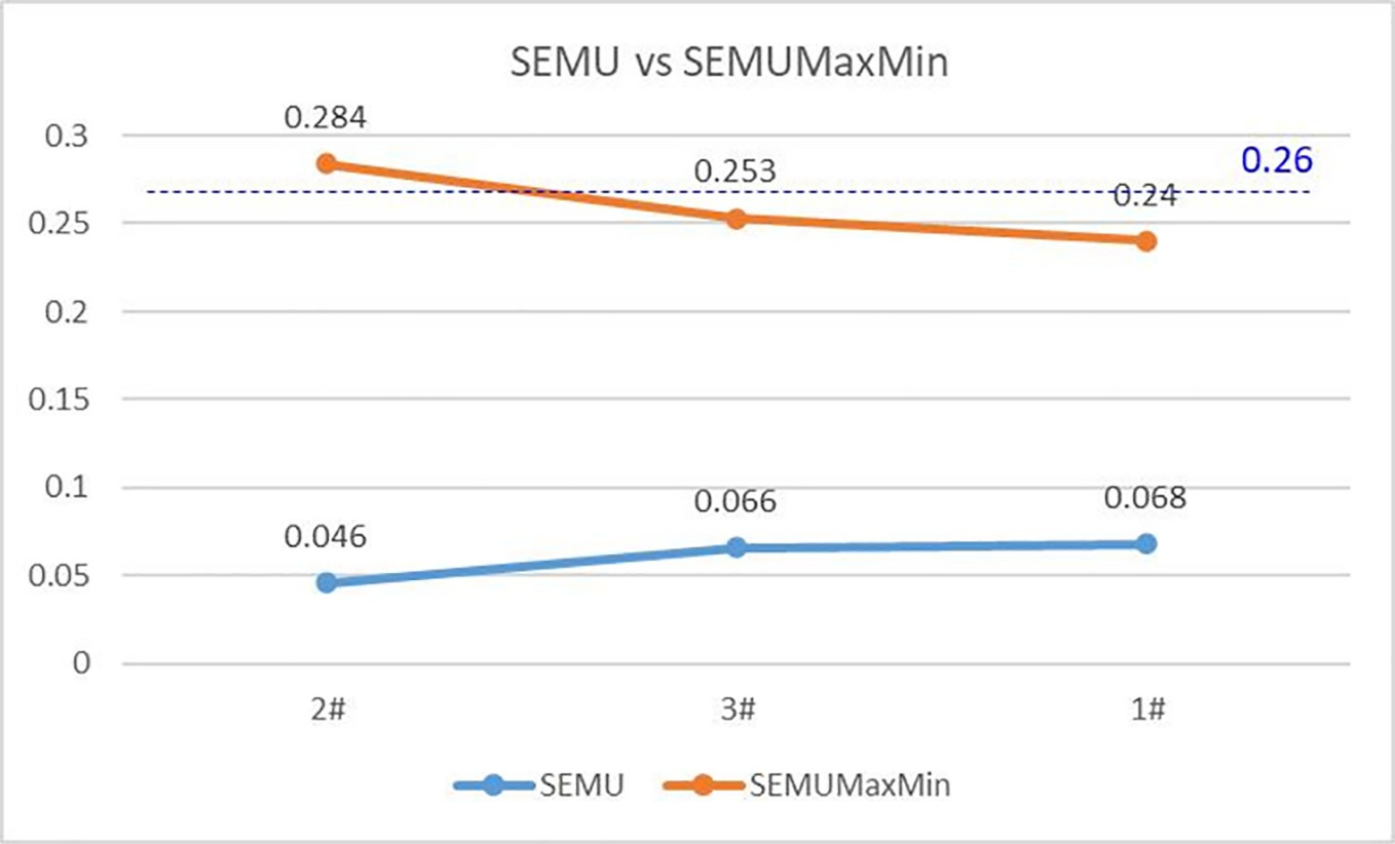
Comparison of SEMU and SEMUMaxMin data.

After obtaining the result of SEMUMaxMin value, this paper sets the threshold value to filter the defects. For example, setting the threshold value to 0.26, this paper filters out the No. 1 and No. 2 defects, and only retains the No. 2 defect with the strongest characteristics as the final defect conclusion data of this image, as shown in Table 3 below. Fig 16 shows that the comprehensive result of SEMUMaxMin is more consistent with the strength of the actual defects.

**Table 3:** Defect results filtered by SEMUMaxMin algorithm.

Defect number	Semu	Semumaxmin	Result
**1#**	**0.068**	**0.240**	**Pass**
**2#**	**0.046**	**0.284**	**Defect**
**3#**	**0.066**	**0.253**	**Pass**

After the algorithm is fully developed, this paper develops the inspection software system, and designs and manufactures the equipment and machines for the mass production operation evaluation on the production site. During the evaluation period, the production line produced a total of 7885 machines, and the evaluated products were 100% rechecked by the quality personnel. After comparing the data, the conclusion was drawn that 69 punctate mura were normally detected by the equipment, 3 were missed, and the visual detection accuracy of punctate mura defects was 96%, of which 2 were missed by SEMUMaxMin card control. It can be detected by adjusting the control parameters, and any missed detection, which is attributed to the reasoning and recognition of the deep learning target detection model, can be identified by tightening the probability filtering threshold of the model. In the subsequent long-term mass production process, the model in this paper also needs to continuously collect defect samples, expand the training set, and continue iterative training to achieve the continuous optimization effect of the accuracy of the model, and optimize the judgment logic of SEMU value according to the engineering detection requirements of the production line, comprehensively improve mura detection performance and mass production adaptation, and help display panel factories improve product yield and production efficiency.

In a recent study [52], authors utilized Convolutional Neural Networks (CNNs) for the detection of mura defects. The proposed research was evaluated on OLED images and identified four types of mura defects. The mura detection accuracy was reported at 86.80%. In comparison, our study achieved a detection accuracy of 96%, demonstrating superior performance in mura fault detection. In the most recent work by Wang et al. [24] a Color-Mura defect detection approach was proposed using adaptive segmentation thresholds. The experimental results from this study indicated an accuracy rate of 87%, which is lower than the accuracy achieved in our current research.

## Conclusion

Aiming at the detection task of weak defects on the surface of organic light-emitting semiconductors, a detection model SmartMuraDetection based on small sample deep learning is proposed in this paper. Firstly, aiming at the detection difficulty of low contrast of surface defects, a gradient boundary enhancement algorithm module is designed to automatically identify and enhance defects and background feature differences. Then, in order to solve the problem of insufficient small sample data set, a Poisson fusion image enhancement module is designed to enhance samples, TinyDetection model adapted to small-scale target detection was constructed to improve the detection accuracy of small-scale target defects. Finally, the SEMUMaxMin quantization module was designed as the post-processing for the result image derived from the network model reasoning, and the accurate defect data was obtained by setting the threshold filtering. The number of small sample set images used in the algorithm experiment was 334, and the trained model was imported into the actual OLED panel production line for mass production evaluation. There are 7885 pcs of mass production operations in the production line, and the visual detection accuracy of point mura defects is 96%, which meets the quality inspection requirements of the actual factory, indicating that this method can effectively detect point mura defects. However, at present, this algorithm is only applicable to the detection of point mura, and there are many types of mura samples in the actual production site, so it is necessary to continuously update and add mura sample data in the future, and optimize the iterative network model and quantization scheme according to different mura forms [53]. In addition, this method can also be used for reference in the detection of weak defects on the surface of other flat plates [54], such as glass surface indentation detection and low contrast pollution detection on the surface of textiles.
